# On the Influence of the Teeth on Tongue Disease

**Published:** 1883-05

**Authors:** W. Fairlie Clarke

**Affiliations:** F.R.C.S., Assistant-Surgeon to Charing Cross Hospital


### On the Influence of the Teeth on Tongue Disease.

BY W. FAIRLIE CLARKE, M.A., M.B., OXON, F.R.C.S., ASSISTANT-SURGEON TO CHARING CROSS HOSPITAL.

Read before the Odontological Society of Great Britain.

MR. PRESIDENT AND GENTLEMEN.—There is hardly a disease of the tongue which may not be either caused or aggravated by an unhealthy condition of the teeth. I hope, therefore, that the subject which I bring before you this evening may not be without interest to the members of the Odontological Society.

A moment's reflection will suffice to show why it is that the influence of the teeth upon the tongue is so great. The tongue is a soft organ, essentially muscular, and making active movements in the performance of all its functions; but it lies in a cavity where it is hedged round on three sides by the teeth. When it is at rest, and the mouth is closed, it is in contact throughout the greater part of its extent with the teeth. They, as it were, fence it in; but, the moment it moves, it is brought into much more active contact with them. Whether its movements are made for articulation or mastication, the tongue is pressed against the teeth, or carried along or beyond them; and in these movements it is liable to be injured by them in many ways. I do not speak of the injuries which the epileptic are liable to inflict upon themselves during their fits, nor of those which the sufferers from tetanus may incur by the spasmodic and forcible closure of their jaws; nor, again, shall I speak of those accidental cases in which the tongue is bitten and lacerated to a greater or less extent. All these are no doubt injuries inflicted by the teeth, but they do not fall within the scope of my subject; nor would there be much profit in dwelling upon them here, for, the teeth being only the instrument by which the wound is inflicted—the real cause having to be sought for elsewhere—such cases fall either under the care of the surgeon as accidents, or under that of the physician as the result of constitutional disease, and are not usually brought under the notice of the dentist.

What I propose to do in this paper is, to review some of the more common affections of the tongue, and to consider how far they are either caused or aggravated by a faulty condition of the teeth, such as the knowledge and manual skill of the dentist could relieve.

What I have to say on this subject will fall under two general heads. First, I shall speak of the ulcerative diseases of the tongue; and, secondly, of its cancerous affections. If I mention what I have learned, or what I have seen, of the effects of the teeth upon these two great classes of tongue disease, and if I allude incidentally to some of the rarer affections of the same nature, I shall probably have touched upon all the points that are of the most interest to my present hearers.

First, then, with regard to the ulcerative diseases of the tongue. These are of several kinds. There are simple or dyspeptic ulcers; there are syphilitic ulcers; and there are aphthous ulcers. Of these, the last, as you are well aware, depend upon the presence of a minute vegetable parasite—the *oidium albicans*—which grows upon and destroys the epithelium. The epithelium falls off and a flat superficial ulcer is left. As this minute fungus is only developed when the general health is much enfeebled—as, for instance, in some infantile diseases, and with adults in the last stages of exhausting complaints, such as phthisis or cancer—the spot thus denuded of its superficial covering rapidly runs on into ulceration. Such an ulcer may no doubt be aggravated by the presence of rough teeth, but they have no share in causing it, nor would it be much improved if they were removed. We may, therefore, dismiss this form of ulceration from our consideration, and turn to others which more immediately concern us.

The other two classes of ulcerative disease that I have named —the simple, or dyspeptic, and the syphilitic—may be determined by some fault of the teeth, and may, to a greater or less extent, be removed by the skill of the dentist. Do not misunderstand me. I do not, of course, say that a syphilitic ulcer, or even a dyspeptic one, can be *caused* by the state of the teeth. No amount of accumulated tartar, no rough edges or sharp points, could effect this. But, given a dyspeptic state of the stomach and of the parts in sympathy therewith, or given that faulty condition of the blood which we call constitutional syphilis, then the presence of a carious tooth may determine the outbreak of an ulcer, or may tend to keep it up and aggravate it. When the general health is good it is remarkable how very tolerant the tongue is of irritations. A whole row of irregular teeth is not enough to produce an abrasion. But let the patient fall into ill health, and then the surface is easily inflamed, and the inflammation quickly runs on into ulceration.

The simple or dyspeptic ulcers are most apt to occur in persons of a full habit of body, who have usually a bright, florid congestion of the tongue, and who habitually eat and drink freely. Such ulcers are generally situated upon the sides and under-surface of the tip, but not unfrequently they are found in the folds of the frænum. It is necessary, therefore, that a careful search should be made for them.

Dyspeptic ulcers are shallow; their bases are flat, and covered with a greyish slough; their edges are rounded and bevelled; they are encircled by an inflamed margin; they are very sensitive to the touch, and painful when the organ is moved; they are often multiple; they run an acute course, and, though successive ulcers may keep coming up for a length of time, no single ulcer lasts more than two or three weeks. Such are the characters of simple or dyspeptic ulcers. But if such an ulcer is brought on (as it very often is) by the irritation of a carious tooth, its appearance and its history may be considerably modified. The importance of examining into the cause of such an ulcer, to see if it depends upon some local source of irritation, is pointed out by Hippocrates, and enforced by Celsus. "When there is a chronic sore on the side of the tongue," says the former, "the surgeon should examine whether it be not occasioned by the sharp point of a tooth." The offending tooth may be either in the upper or lower jaw. Sometimes a rough edge in the lower jaw irritates the tongue as it lies at rest. Sometimes it comes in contact with a sharp point in the upper jaw in every movement that it makes. At first that portion of the tongue which is irritated by the offending point becomes more vascular than usual, of a brighter red, and the papillæ are somewhat enlarged. If the irritation continues a slight circumscribed swelling arises, and subsequently the tissue softens, and a small ulcer is the result. After the irritation has lasted for a length of time, there may be more or less induration, which not unnaturally excites the fears of the patient. If appropriate treatment be not at once adopted, such ulcers get rapidly worse. They become foul, excavated, with irregular callous edges, and a hardened base. The tongue is furred, the breath is offensive, and it is evident that there is considerable derangement of the stomach. It is painful to move the tongue, and on this account speech is interfered with. If such an ulcer be neglected, it may lead to the most serious consequences, even determining the seat of a cancer. When there is a large, spreading ulcer in the tongue, when the lymphatic glands at its root are becoming indurated and enlarged as the result of the acute inflammation, and when the patient's general health is failing, he must be considered to be in a position of great jeopardy. On the treatment which is then adopted it depends whether he is restored to health, or whether the disease assumes the clinical features of a malignant sore.

Leaving the dyspeptic ulcers, I now pass on to those which are of syphilitic origin. Syphilitic ulceration of the tongue may either be deep-seated or superficial. The deep ulceration is brought about by the softening and discharge of a gummy tumour in the substance of the organ; the superficial is commonly determined by some local source of irritation. The vertical cracks and fissures, which are so often seen on the sides of the tongue in those who have constitutional syphilis, follow the course, I imagine, of the folds into which the mucosa falls during movement; just as we often see eczematous rashes about the folds of the neck, or of the thighs, in infants. And in a similar way any other habitual pressure may lead to a superficial ulcer, and among such none is more common than the irritation caused by sharp or rough teeth, or by the fixings of artificial teeth. Here, then, we come once more in contact with the proper subject of this paper. The ulceration may remain superficial, of rather a chronic and indolent character, but exquisitely sensitive, so that the least touch causes the patient acute pain; or the base may become indurated, the surface excavated, the surrounding tissue inflamed, and the glands beneath the jaw enlarged. Then the case approaches in appearance to the deep form of ulceration, arising from the discharge of a gummy tumor. Cases such as I have described, in which there is an excavated ulcer with thickening of the surrounding tissues, enlargement of the adjacent glands, and general cachexia, are sometimes very difficult to distinguish from cancer, and call for the most careful study on the part of the surgeon.

We may now consider the treatment of these various ulcerative affections; for the same general principles which are applicable to the dyspeptic ulcer are applicable also to the syphilitic. These principles are: first, rest—physiological and mechanical rest; second, proper local applications; third, suitable constitutional treatment.

But how are we to procure rest? First and foremost, by removing any local source of irritation; and here it is that the aid of the dentist is so valuable for the successful treatment of ulcers of the tongue; and it is upon this point more particularly that I hope to learn much from the members of the Odontological Society. Given an ulcer of the tongue, which is either clearly due to, or probably kept up by, the irritation of bad teeth, what is the best plan to adopt? Should such teeth be removed altogether, or should they be filled up or filed down? Or has the progress of modern dentistry devised any plan which may be better than either? These questions I shall not attempt to answer myself. It would ill become me, before such an audience as this, to express any opinion. But I hope that they will be answered hereafter by those who are present, and that they may serve to draw forth the experience of the members of the Odontological Society with regard to tongue disease.

The local treatment of ulcers of the tongue may be summed up in a few words. The surface of the sore should be carefully, but thoroughly, touched with lunar caustic, and a soothing or cleansing mouth-wash prescribed. Nothing is better than a solution of chlorate of potash. At the same time the patient should be requested to abstain as much as possible from talking for a few days, until the ulcer has cicatrized. I find the diluted nitrate of silver (equal parts of nitrate of silver and nitrate of potash) very suitable for these cases. It is quite as potent a caustic as is required; it is less painful in its application than the pure nitrate of silver; and it is so hard that it can be cut to a fine point, and introduced to the bottom of cracks and fissures.

With regard to the constitutional treatment, it is of the first importance that the patient should live entirely upon a bland and fluid diet, avoiding all hard pieces of meat or bread, all pungent condiments, and everything that can irritate or inflame the morbidly sensitive organ. At the same time we must take care that he has plenty of nourishment; he must not be allowed to fall below his usual standard of health. Some patients have such a dislike to be fed upon what are called *slops*, that it requires a good deal of tact and judgment on the part of their friends to get them to take their accustomed amount of nutriment in this form. Having laid down positive orders with regard to the general character of the diet, we then ask what can be done by medicines? If the ulcer is of the dyspeptic kind, we must first give alteratives—a little grey powder or compounded rhubarb powder, to stimulate the liver, and regulate the bowels, and this we speedily follow up with tonics. Among this class of drugs, none are better suited to the present purpose than the mineral acids and the preparations of bark. If the ulcer has a syphilitic origin, then a mild mercurial course, or the iodide of potassium, must be given in order to effect a cure. If the case is one of those in which the diagnosis is uncertain, and there is a serious fear of cancer, it is well to bring the patient speedily, but slightly, under the influence of mercury, and then two or three weeks will suffice to show whether any benefit may be expected from antisyphilitic remedies.

I pass on now to the second great class of diseases with which I said I should deal, namely, the cancerous.

Cancer of the tongue is a common, and a very fatal affection. I have shown in my "Treatise on the Diseases of the Tongue," that, among surgical cancers of separate organs, it stands second in point of frequency, the first place being occupied by those of the breast. With regard to the fatality of lingual cancer, I have demonstrated, by comparing a large number of cases, that its average duration is only fifty-seven weeks, *i.e.*, somewhat less than fourteen months. I have, however, also shown that by judicious and timely interference by means of an operation, this period may be very considerably extended, and the condition of the patient moreover be rendered much more tolerable. To effect a cure is, I fear, almost beyond our power.

Cancer of the tongue begins either as a hard lump deeply placed in the substance of the organ, or as a pimple or vegetation on its surface.

When it begins superficially, it is worthy of notice how often its origin may be traced to some local irritation, such as the jagged edge of a tooth or the smoking of a short clay pipe. Indeed, there is no class of cancers which affords a stronger argument in favor of the local origin of malignant disease than these cancers of the tongue. If we could follow them all up to their source, I have no doubt we should find that the great majority occur in tongues that are already damaged from some cause or other.

When cancer has once attacked the tongue, no matter what may have been the exact mode of its invasion, its march is characterized by symptoms which are common to all its varieties. There is occasional darting pain, radiating towards the ear, temple, and vertex; the diseased portion is tender; eating is rendered difficult; speech is thick and indistinct; the base of the tongue becomes infiltrated, and the organ cannot be moved freely or protruded; it seems as if it were bound down to the floor of the mouth. The sublingual and submaxillary glands, as well as those which are connected with the lymphatic system, become enlarged and painful. There is an increased flow of saliva. The circulation through the brain is disturbed, and the patient complains of giddiness and headache. The cachexia now becomes more marked. Rapid wasting and loss of strength manifest themselves. The local disease gradually involves more and more of the tongue. Sometimes large sloughs occur in consequence of pressure upon the lingual artery or its branches, and profuse bleeding may take place. The difficulty of swallowing, and even of breathing, is great, on account of the obstruction which the disease causes at the pharynx; and this obstruction is increased by œdema, the result of retardation of the venous current. Sometimes the œdema is so great that the tongue is protruded from the mouth, and cannot be retracted. Gradually the growth invades the neighboring parts, and shows itself in the tonsils, the gums, and some other situations. Frequently there are affections of the air-passages—*e.g.*, bronchitis or pneumonia—and the scene closes upon one of the most painful cases that it is the duty of the surgeon to attend.

It is noteworthy, particularly in connection with the special subject of this paper, that cancer of the tongue always begins at or near one side of the organ. I have never seen a case (unless it arose out of that strange and rare disease *ichthyosis linguæ*) which did not fall under this rule. Indeed, so constant is it that it is taken as an aid to diagnosis when there is any difficulty in establishing the precise nature of the case. And sometimes in the early stage it is no easy matter for the surgeon to be certain with what he is dealing. A commencing cancer may be not unlike a simple hyperplasia, or a dyspeptic ulcer, or even a mucous tubercle. Nay more, any of these morbid states, which we are accustomed to regard as being in themselves far removed from epithelioma, may be so aggravated by local sources of irritation that in time they become truly malignant diseases. This is not the place to discuss the conversion of simple into malignant sores, and it would carry me beyond my proper subject if I were to do so. I may merely state my confident opinion that such a change often happens, and that sores which are in their origin innocent, and which might by judicious treatment have been brought to a favorable issue, become malignant by protracted irritation and maltreatment.

Now, if cancer of the tongue be thus fatal, and if simple affections can be so irritated as to become malignant, how very important it is to remove such sources of irritation; and it is to effect this that the surgeon so often has to call in the aid of the dentist. When there is the slightest suspicion as to the nature of a sore on the tongue, there ought to be no hesitation, no half-measures. Any irregular teeth that may be at fault should be at once removed, and artificial teeth should be laid aside for a time. The patient should be ordered to abstain as much as possible from speaking, and to live entirely upon a liquid and bland diet. At the same time such medicine as the character of the sore may suggest should be prescribed—perhaps iodide of potassium, perhaps mercury, perhaps mere alteratives or tonics. If this line of treatment be pursued, it will soon be apparent whether the aid which the dentist can afford is of any avail, or whether the case must be dealt with by the operating surgeon.

Thus have I laid before you the commonest affections of the tongue which bring your branch of surgery and mine into contact. Other diseases of the tongue I have seen and read of, such as ichthyosis or neuralgia, which may depend upon a carious state of the teeth, and the nervous impression conveyed therefrom. But these are comparatively rare affections, while those with which this paper is chiefly occupied are very common. I hope, therefore, that in the course which I have followed, I have chosen that which is most likely to lead to practical results, and to make this evening both pleasant and profitable by the mutual interchange of our experience.

